# Hydro-mechanical coupling characteristics and weakening mechanisms of filling joint resulting from water injection

**DOI:** 10.1038/s41598-022-26308-6

**Published:** 2022-12-19

**Authors:** Yixin Liu, Chuanhua Xu, Jiang Xu, Xuemin Zeng

**Affiliations:** 1 State Key Laboratory of Safety and Health for Metal Mine, Maanshan, 243071 China; 2 College of Resources and Environmental Engineering, Jiangxi University of Science and Technology, Ganzhou, 342399 China; 3 College of Safety and Environmental Engineering, Shandong University of Science and Technology, Qingdao, 266590 China; 4 State Key Laboratory of Mining Disaster Prevention and Control Co-Founded By Shandong Province and Ministry of Science and Technology, Shandong University of Science and Technology, Qingdao, 266590 China; 5 State Key Laboratory of Coal Mine Disaster Dynamics and Control, Chongqing University, Chongqing, 400044 China

**Keywords:** Civil engineering, Natural hazards

## Abstract

The injection of fluids into fault gouges in rock formations disturbs the in situ stress conditions, leading to fault slip and increasing the risk of inducing earthquakes. The weakening effect and the permeation of the injected fluid can be influenced significantly by the presence of fault gouges. To investigate this issue, the hydro-mechanical characteristics of fault gouges were evaluated using physical tests to study the combined effects of coupling injecting water and shear deformation. We propose a new experimental apparatus that allows us to measure the spatial distribution of the thickness of a gouge layer sample under combined conditions of shearing and water injection, using 3D scanning technology to evaluate the primary flow path. The test results showed that injecting water had a significant effect in reducing the maximum shear strength, but the degree to which the strength was affected varied with the gouge fill material. The effect of shear deformation is that it will increase the inhomogeneity of the thickness distribution and therefore the distribution of injected water along the fault is not uniformly radial. Additionally, the properties of gouge fill material have an important influence on flow characteristics during fault slipping.

Hydro-mechanical coupling instabilities that have been observed to have occurred in unusual locations have become a hot topic of discussion in the world, as a result of the concern that certain types of industrial activity may cause damaging hazards^1–4^. In particular, the injection of fluids into underground formations^2,5–7^ and deep-level mining^8,9^, represent two situations that have changed in situ stress fields (see Fig. 1), and have been identified as particular activities that may induce earthquakes. The other necessary component for an induced instability is the existence of faults in the ground formations^10–12^. Fluid injection into underground formations is likely to cause dilation of that strata and will increase *in-situ* stress. In contrast, deep-level mining may result in a decrease in *in-situ* stress. Both of these cases can cause slip to occur in nearby faults. Due to formation disturbance or fault slip, the permeability of the rock strata increases, which allows the injected fluid or groundwater to penetrate deeper into the fault, lubricating the joint and leading to a larger displacement of the fault slip through the coupling of the water with fault gouge, finally inducing an earthquake^5,10,11,13,14^. The basic physical mechanism for inducing seismicity is well understood in terms of the effective stress principle^15^:

1 $$ \sigma^{\prime } = \sigma_{N} - P_{p} $$

where $\sigma^{\prime }$ is the effective normal stress, *σ*_*N*_ is the tectonic normal stress, and *P*_*p*_ is the pore fluid pressure. However, considering a simple Coulomb model for frictional strength^16^:

2 $$ \tau = C + \mu \sigma^{\prime } $$

where *τ* is shear strength, *C* is cohesion, and *μ* is the coefficient of internal friction. The Coulomb failure relationship of Eq. (2) predicts that the stability of frictional sliding is dependent upon *C*, *μ,* and *P*_*p*_, as is illustrated in Fig. 2.

**Figure 1:**
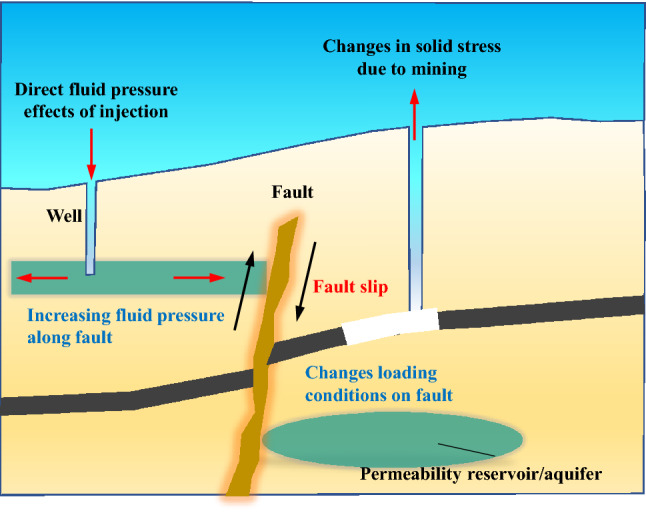
Schematic diagram of mechanisms for inducing earthquakes. An earthquake may be induced by increasing the fluid pressure acting on a fault (left) or by changing the shear and normal stress acting on the fault (right).

**Figure 2:**
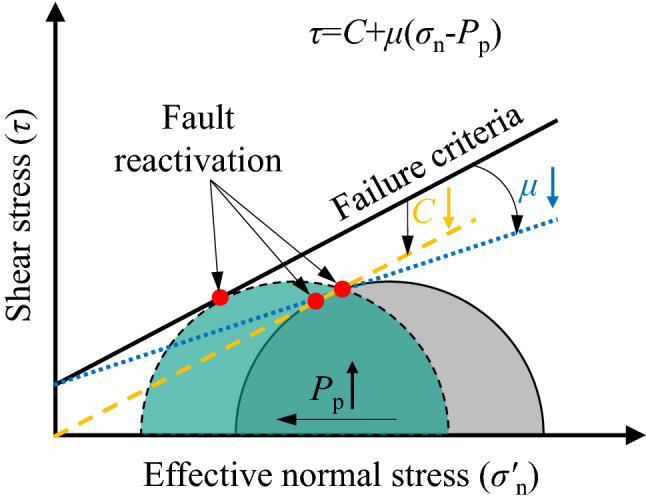
Mohr–Coulomb diagram for shear failure.

As is shown in Fig. 2, as fluids at high pressure infiltrate the fault (*P*_*p*_ ↑), the effective normal stress of the stress condition along faults before fluid injection (grey semi-circle) is reduced, favoring the conditions for fault reactivation (green semi-circle)^2^. In addition, due to the physical and chemical interaction coupling the water and the fault gouge, the cohesion (*C*) decreases (yellow line) and the coefficient of internal friction (*μ*) decreases (blue line) also favoring the conditions for fault reactivation (red dot). Wang et al.^17^ stated that water affects the intrinsic property of the clay particles by changing the state of hydration of the ions on the surfaces of each clay particle, thereby modifying the contact points or bonding between them. Ma et al.^18^ investigated the evolution behavior of overall permeability that can result from small particle migration from the granular sandstone at different original porosities, particle size compositions, and water flow pressures. Takahashi^18^ further found that the gouge can seal faults by grain comminution, as the smear forms a membrane seal by entraining in a primary unit with low permeability. Faulkner et al.^19^ proposed that clay-bearing fault gouge is one of the lowest permeability fault rocks and it plays a critical role in mitigating fluid loss. Keulen et al.^20^ suggested that hydrostatic conditions can be caused by cementation, i.e., the filling of pore spaces by precipitation of material from the fluid. Overall, the mechanical behavior from injection of the coupling fluid and the associated fault slip is closely related to the joint asperity^21,22^ and specific fault gouge materials^23–26^. However, studies reported in the literature have focused mostly on the permeability and migration characteristics of the fault gouges, but have ignored the coupling mechanism of fluid injection and gouge deformation due to fault slip^27^. The mechanism relating fluid pressures to fault weakening and fault rupture still remains unclear^5,28^, especially for large-displacement faults^10^.

Rohmer et al.^12^ summarized four deformation mechanisms of fault gouges: (1) disaggregation of grains via grain boundary sliding and reorganization, and cement grain breaking or granular flow with little to no influence on porosity; (2) cataclasis involving grain abrasion and fracturing to different degrees, which commonly reduces porosity; (3) phyllosilicate smearing, where clay minerals are abundant and make barriers to flow along the bands; (4) dissolution and cementation, which are mostly post-deformation and would have additional impact on band porosity. In the present study, to take into account the deformation stage of fault gouge, three materials including fine sand, clay and gypsum were selected. A series of laboratory direct shear tests under fluid injection on rough gouge-filling fractures was performed to simulate the hydro-mechanical characteristics of coupling fluid injection and fault-slip. A new method was proposed to measure quantitatively the gouge layer spatial thickness distribution (GLSTD) and investigate conceptually the evolution of the flow path during shear processes.

## Method

### Material and sample preparation

**Figure 3:**
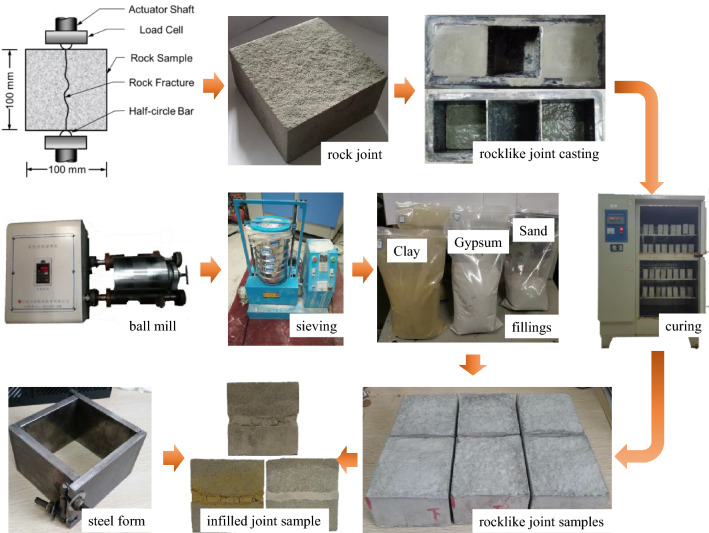
Sample preparation procedure.

It has been proved that shear slip and the failure of discontinuities are responsible for rock mass instability and geological disasters^29,30^. Traditionally, tests on idealized joints (e.g., triangles and trigonometric curves) have been conducted, due to difficulty in collecting natural rock joints^30,31^. However, it has been observed that man-made fractures can be acceptable substitutes for natural fractures if the spectral and statistical properties of natural joints are analyzed^32^. In the present study, a modified Brazilian method^33–35^ was applied to produce a fresh, exactly mated tensile fracture interface in sandstone samples (as shown in Fig. 3). The artificial sandstone joints were replicated by a mixing ratio of 2:3:1 by weight of R42.5 ordinary Portland cement with fine sand and water. First, the lower half of a joint sample was placed in a steel former. The cement(-sand) mortar was poured into 100 × 100 × 100 mm^3^ rectangular molds for casting the joints. Finally, the cement mortar joint samples were transferred to a cement maintainer for a 28-day curing period. The density of the samples was 2.05 g/cm^3^, the uniaxial compressive strength was 77.57 MPa, the cohesive force was 14.37 MPa, the internal friction angle was 62.39°, the elastic modulus was 5.95 GPa, and the Poisson ratio was 0.18. A hole with 8 mm diameter was drilled in the center of the samples as the water injection hole.

To take into account the deformation stage of fault gouge: fault weathering and grain cement breaking produced fine grain materials. Grain abrasion and phyllosilicate smearing produced clay minerals and cementation during post-deformation. Accordingly, three artificial materials including fine sand, clay, and gypsum were selected as infilling gouges. The particle size was < 0.15 mm and was selected by using a standard dry sieve array. Before forming, the filling materials were mixed with water in a mixing ratio of 10:3 by weight, and the mixture was filled between the upper and lower halves of joint samples. Finally, the gouge layers were molded with layer thickness of 3 mm by controlling the displacement of a load cell with a steel former, and the samples were dried in a ventilated area for two days.

### Test apparatus

**Figure 4:**
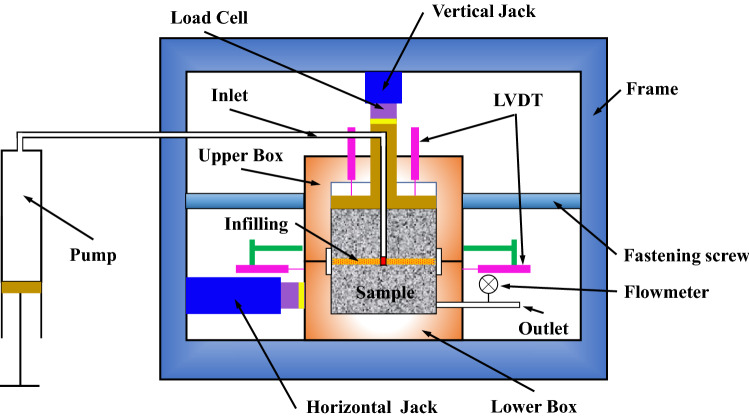
Digitally-controlled shear-flow testing apparatus.

Laboratory tests were performed using a direct shear-flow test apparatus under constant normal load, see in Fig. 4^36^. The test apparatus mainly consisted of two units: a controlling part and a loading part. The controlling part was monitored and produced a continuous data record of mechanical behavior, including force and displacement. The loading part had a pair of shear boxes, a water injection pump, load cells, and linear variable displacement transducers (LVDT). As shown in Fig. 4, the height of the upper shear box was significantly higher than the sample to allow for shear dilation. The upper box was fixed by four fastening screws. Four LVDTs were distributed evenly on the normal pressure plate to record normal displacement, and two LVDTs were applied to record the shear displacement. The shear and normal force are applied through a hydraulic jack and were calibrated by a load cell. The water pressure was provided through a water pressure pump that was similar in action to a syringe. The water was injected through the water inlet under constant pressure, and the flow rate was monitored using a flowmeter in the water outlet.

All infilled jointed samples were sheared at a constant rate of 1 mm/min. Before applying the shear force, the normal force was loaded at a rate of 0.2kN/s until it reached 30kN and then remained constant. The water injection pressure was loaded and held at 0.3 MPa. Based on the preliminary tests, measurement of the residual shear strength of the fault gouge required a relatively small shear displacement^35,37^, and the value of maximum shear displacement was selected to be 10 mm.

### Method of calculating the thickness variability

**Figure 5:**
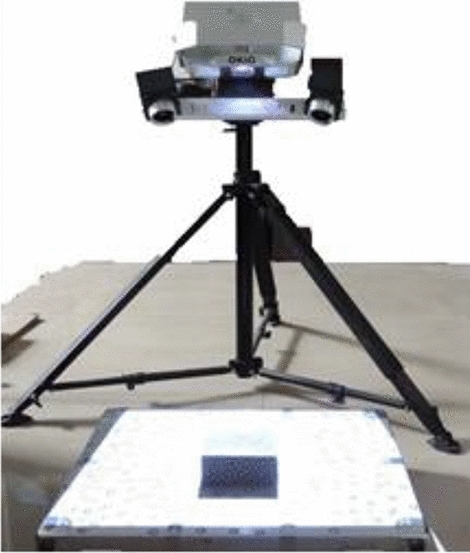
3D scanning device for rock structure surface.

A 3D scanner (the brand is Tianyuan-OKIO-B) was applied to digitize the morphology of the surface, see in Fig. 5, and the 3D scan system projected a series of structured white-light fringe patterns onto the surface of the joint. Two CCD cameras were employed to capture images of these patterns automatically^38,39^. The measurement accuracy of the three-dimensional scanner is 0.02 ~ 0.01 mm. The average point distance of 3D scanner is 0.15 ~ 0.07 mm. The scanning measurement results can output ASC point cloud file format, and Kriging interpolation reconstruction is performed at an interval of 0.2 mm.

**Figure 6:**
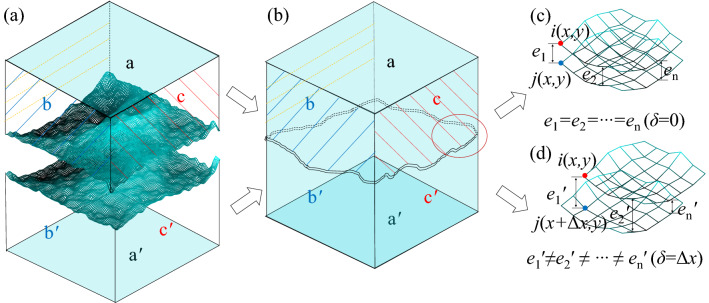
Calculation process for GLSTD. (**a**) upper and lower halves of the joint sample obtained by 3D scanning, (**b**) matching the upper and lower halves together for 3D scanning, (**c**) initial GLSTD, and (**d**) GLSTD at shear displacement (*δ*) is Δ*x*.

To calculate the GLSTD, a new method was proposed, based on three-dimensional scanning technology. The calculation process of GLSTD can be described as follows: First, the 3D surface profiles of the upper half and the lower half then are obtained using the 3D scanner, respectively (see Fig. 6a). Then, the upper half and the lower half of a cement mortar joint sample are matched together and the external 3D profile of the whole sample is obtained using the 3D scanner (see Fig. 6b). Finally, using an automatic recognition and fitting technique (selecting the upper half and the three same surfaces of the whole sample as the datum plane respectively, as shown in Fig. 6, where plane a and plane b and plane c are selected as the datum planes), the upper half and the lower half are matched into the whole sample. By this method, the initial GLSTD can be obtained by extracting the three-dimensional data for the upper and lower joint surfaces, which can be calculated by:

3 $$ H_{{\left( {x,y} \right)}} = Z_{i(x,y)} - Z_{j(x,y)} $$

where *H*_(*x*, *y*)_ is the thickness at point (*x*, *y*), *Z*_*i*(*x*, *y*)_ is the initial height of the upper surface at point (*x*, *y*), *Z*_*j*(*x*, *y*)_ is the initial height of the lower surface at point (*x*, *y*), see Fig. 6c. When shear displacement occurs, by monitoring the shear displacement and the normal displacement of the sample, the change in the tangent and height of the joint surface data are calculated as follows:

4 $$ H_{{\left( {x,y} \right)}}^{\prime } = Z_{i(x,y)} + \Delta v_{{\left( {x,y} \right)}} - Z_{j(x + \Delta x,y)} $$

where $H_{{\left( {x,y} \right)}}^{\prime }$ is the thickness at point (*x*, *y*) with shear displacement *δ* is Δ*x*, *Z*_*i*(*x*, *y*)_ is the initial height of the upper surface at point (*x*, *y*), Δ*v*_(*x*, *y*)_ is the normal displacement of the upper surface at point (*x*, *y*), and *Z*_*j*(*x*+Δ*x*, *y*)_ is the initial height of the lower surface at point (*x* + Δ*x*, *y*). The GLSTD during the shear process is obtained (see Fig. 6d: the average thickness *E* is equal to the average value of the sum of all points of thickness).

## Results

### Mechanical behavior and permeability

The relationship between the transmissivity and the flow rate of radial flow from the injection hole is given by^40,41^

5 $$ Q = \frac{{2\pi T\left( {h_{i} - h_{0} } \right)}}{{\ln \left( {r_{0} } \right) - \ln \left( {r_{i} } \right)}} $$

where *Q* is the flow rate, *T* is the transmissivity, *h*_*i*_ is the head on the inner surface with radius *r*_*i*_, and *h*_0_ is the head on the outer surface at radius *r*_0_. It can be found that the flow rate is proportional to the transmissivity when the other parameters remain unchanged. However, the effective permeability radius is unstable with the change of the fluid flow path during the process of shear deformation, see in Fig. 10. In the present study, in order to measure permeability more accurately, the flow rate was measured directly and was applied to represent the fluid migration process.

**Figure 7:**
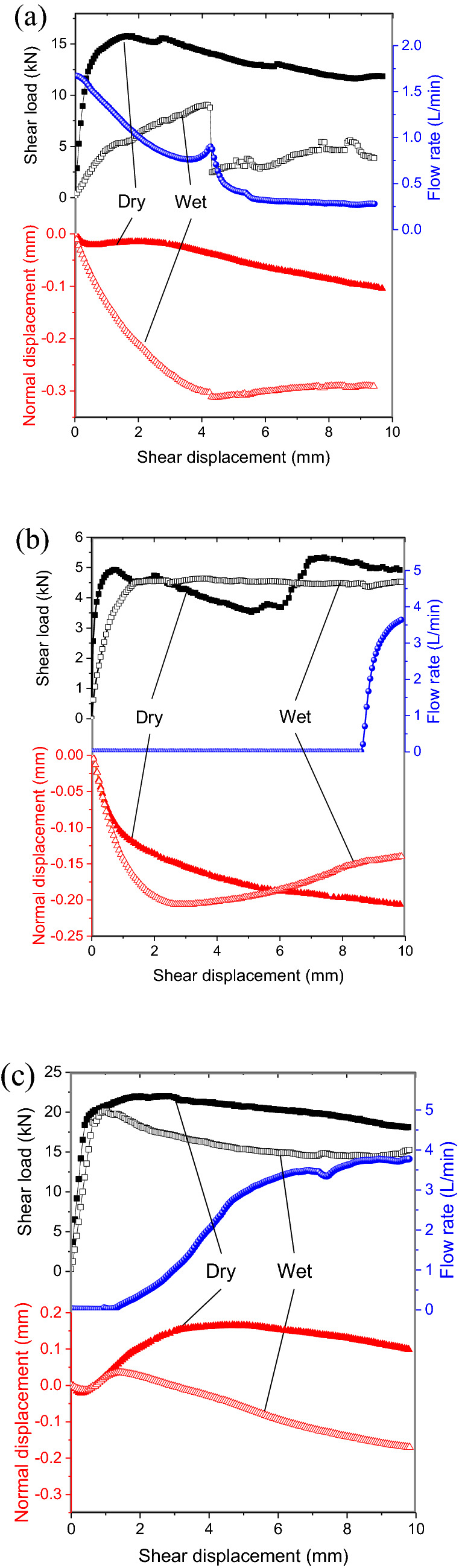
Mechanical behavior with fill materials of (**a**) sand, (**b**) clay and (**c**) gypsum.

The results of the mechanical tests described hereafter are shown in Fig. 7. For all cases studied under dry conditions (without injecting water), the shear load increased steeply with shear displacement before the commencement of sliding. Thereafter, they exhibited stable sliding for the range of shearing investigated. On the other hand, different filling materials with or without water injection showed different weakening behaviors.

The mechanical behavior under a sand layer filling is shown in Fig. 7a. It was found that injecting water (wet condition) had an obvious weakening effect on the shear strength. It is worth noting that a marked reduction in the shear load occurred when water was injected at a shear displacement *δ* = 4.242 mm. Correspondingly, the flow rate increased slightly before that point and continued to decline beyond it. It can be inferred that, during the initial stage, under the effect of the compression-shear load, the sand layer is compacted and the porosity decreases, leading to the decrease in the flow rate^19^. This could be speculated that when the shear displacement reached 4.242 mm, the sand particles accumulate in the region with the smallest thickness under the hydraulic pressure, which limits any further change in thickness and this forms a local stress concentration. With the continued increase in shear displacement, the sand particles are crushed or broken^42^, and the stress concentration instantly lifts, resulting in a sharp decrease in the shear load. This can be also obtained from the normal displacement, and it decreases very clearly when the shear displacement is 4.242 mm. It was found that the normal displacement under the condition of water injection was significantly lower than that under the dry condition, which is maybe due to the decrease in the friction between sand and fracture surface and between sand particles, and that promotes the ease of flow.

The mechanical behavior under a clay filling layer is shown in Fig. 7b. It was found that the shear strength decreased slightly under the condition of water injection, and the change was stable after the peak value. However, the normal displacement exhibited a clear difference. In the initial stage of shear displacement loading, the decreased rate of normal displacement with water injection was higher than was that under dry conditions. The normal displacement under dry condition continued to decrease and the normal displacement with water injection showed a tendency to cause dilation of the gouge material after reaching the lowest displacement value. We speculated that this was caused by a decrease in the effective stress and that the clay was swelling due to the infiltration of water^17^. It should be noted that the flow rate showed a significant rise when the shear displacement reached 8.426 mm, and before that the flow rate was zero. Due to the good sealing and deformation capability of the clay, it will be deformed continuously to adapt to the new thickness distribution during the shearing process. However, when the shear deformation is large, the clay seal layer is broken^43^, and a large void will appear locally, which tends to form the flow channel^44^, and then the flow rate increases and it expands the flow channel by further eroding the clay.

The mechanical behavior with a gypsum gouge fill layer is shown in Fig. 7c. It was observed that, similarly, the shear strength and the normal displacement with water injection exhibited significant weakening. However, before flow occurs, the variation in the shear load and the normal displacement with shear displacement when injecting water are consistent with the behavior under dry conditions. By contrast with a clay fill layer, when the shear displacement was only 1.328 mm, flow occurred and continued to increase as the shear displacement increased. With an increase in flow, the weakening effect on mechanical behavior became progressively more evident, especially in the case of the normal displacement.

### The DLTD and damage to gouge layers

**Figure 8:**
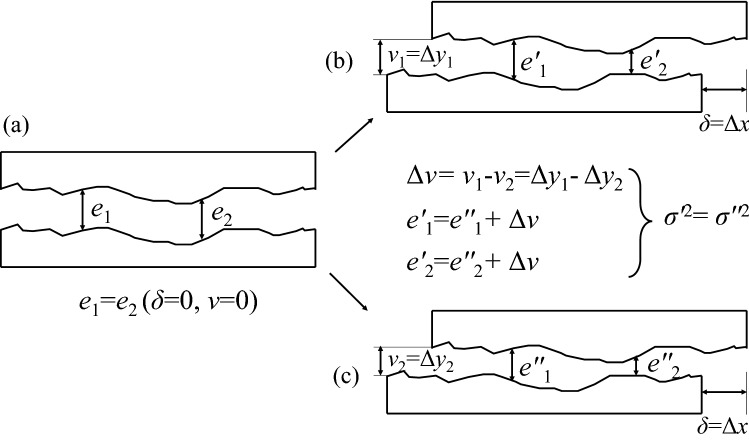
Conceptual diagram of the calculation of the thickness variation under different normal displacement.

**Figure 9:**
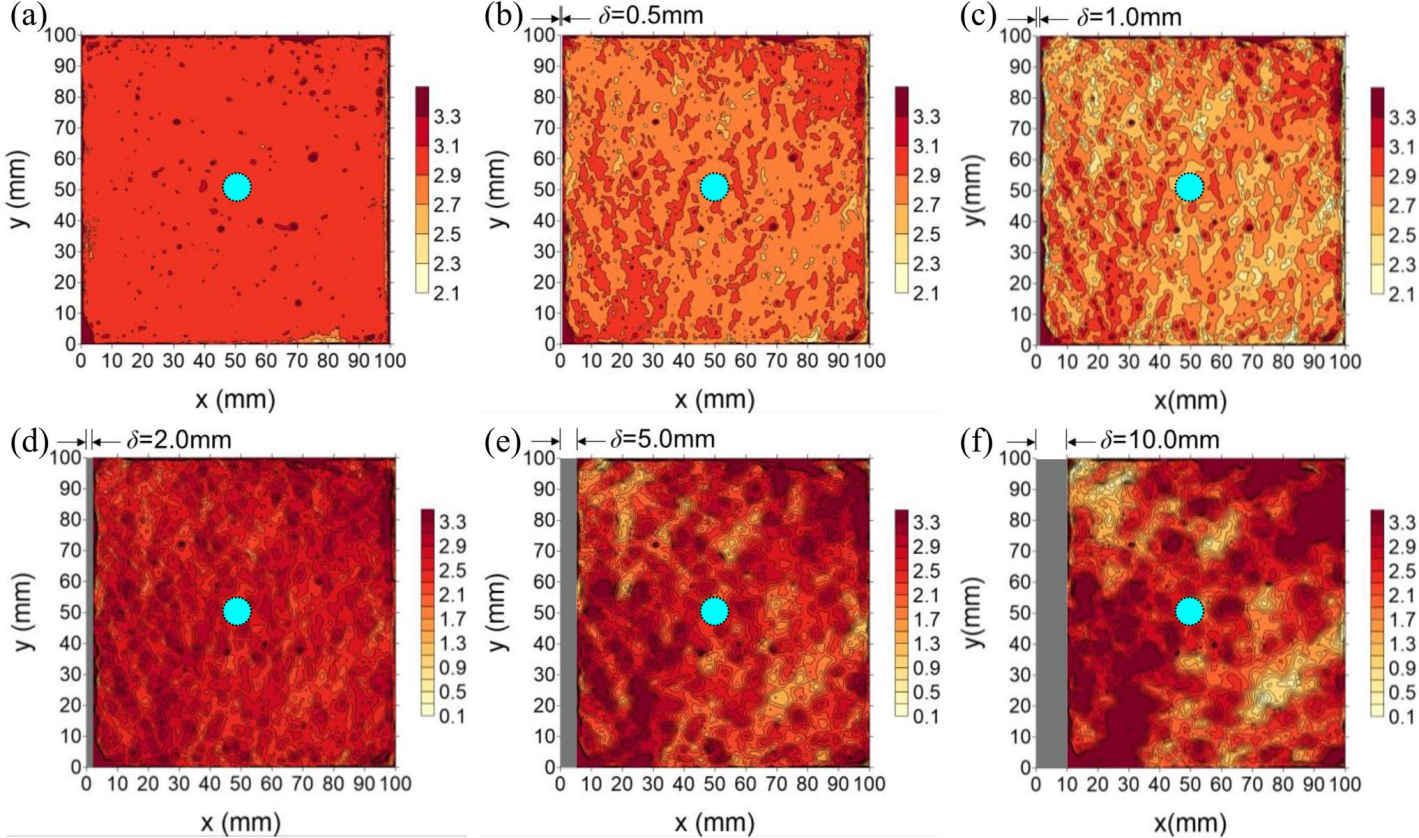
Evolution of thickness distribution with shear displacement and a sand fill layer (“dashed blue” is the water injection hole), (**a**) *δ* = 0 mm, (**b**) *δ* = 0.5 mm, (**c**) *δ* = 1.0 mm, (**d**) *δ* = 2.0 mm, (**e**) *δ* = 5.0 mm, (**f**) *δ* = 10.0 mm.

Before analyzing the changes in gouge layer thickness under different conditions, the influence of normal displacement was considered. As it is known that the samples used in the present study came from the same artificially mated tensile fractures, at the initial stage, the shear displacement (*δ*) and the normal displacement (*v*) both were zero, and the thickness of the different positions was equal (see Fig. 8a). When shear displacement occurs, the relative dislocation position of the upper and lower parts of the samples is the same under same shear displacement and the only difference is in the normal displacement (see Fig. 8b,c). However, it was observed that the difference in normal displacement only affected the average thickness and did not change the degree of dispersion or any inhomogeneity in the thickness distribution, that is, the variance in the thickness (*σ*^2^) was unchanged (see Fig. 8). Therefore, only one case with a sand fill layer under water injection was selected during the present study, in order to analyze the variation in thickness during the process of shear deformation (see Fig. 9).

**Figure 10:**
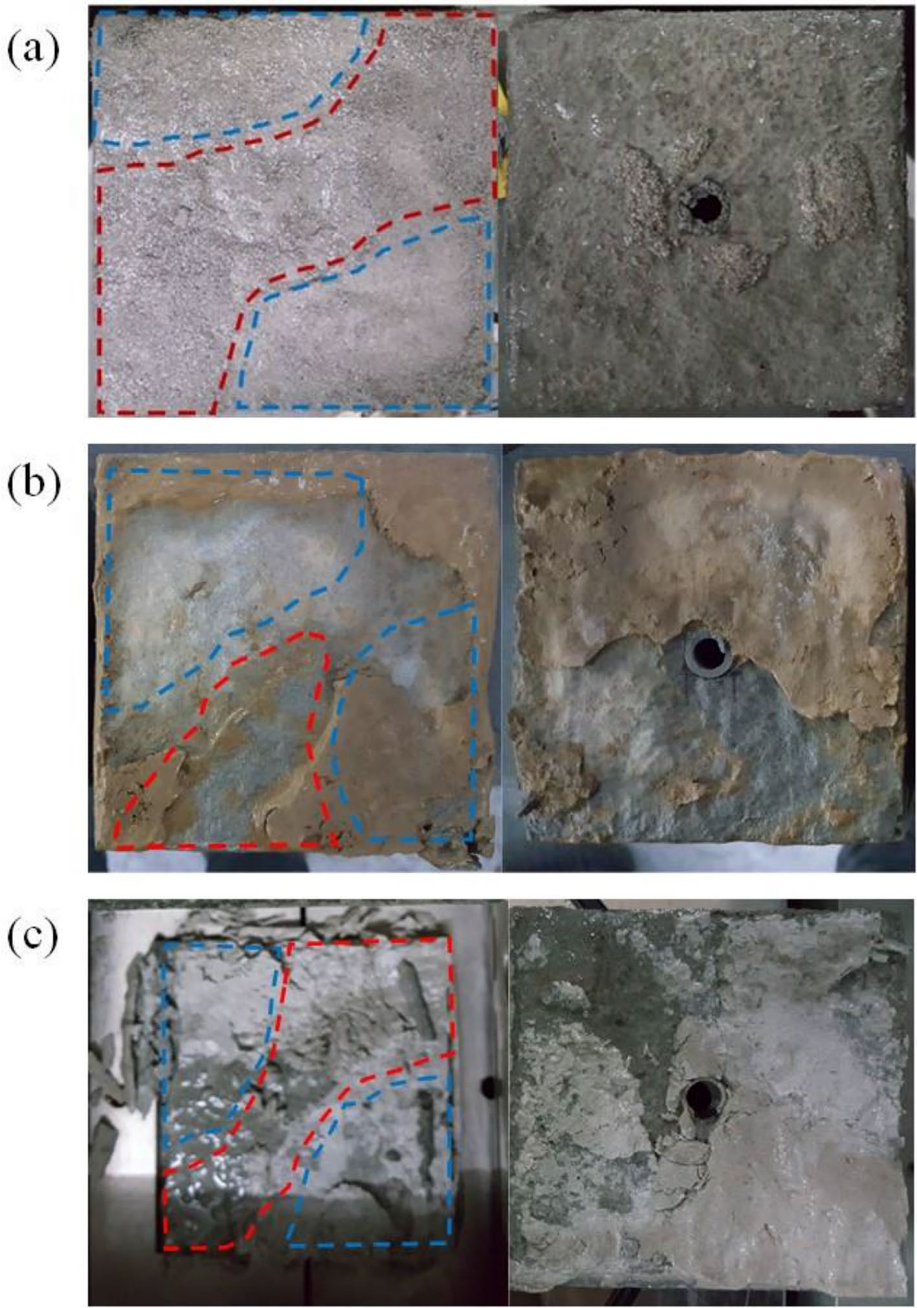
Damage to the fill layer after shear deformation for different materials: (**a**) sand, (**b**) clay, (**c**) gypsum. The images which feature a hole are the upper halves of the samples. (“dashed red” are main water flow regions, “dashed blue” are main stress concentration regions).

As shown in Fig. 9, at initial stage, with a shear displacement *δ* = 0 mm (Fig. 9a), the thickness of every point is almost equal (the difference at the point of dispersion is caused by bubbles in the cement mortar during pouring). However, when shear displacement occurs, even when small (Fig. 9b), the thickness distribution exhibits a difference and it becomes progressively more evident with the increase in shear displacement. When the shear displacement reaches 10 mm, the area with large gouge layer thickness is shown in red and the area with narrower gouge layer thickness is shown in yellow area, see Fig. 9f. From this, it can be inferred that the larger thickness regions are the main flow channel, while the smaller thickness regions are the main stress concentration region. This also indicates that water injection is not uniformly radial along the fault^41^, especially for large fault-slip conditions. Figure 10 shows the damage to the fill layer after shear deformation for different fill materials. It is evident that the water flow regions and the stress concentration regions can be clearly distinguished. For a sand fill layer (Fig. 10a), the sand particles are clear to be seen in the fluid flow regions, while the stress concentration regions are turbid, which is caused by sand grain boundary sliding, crushing and breaking^12,45^. For a clay fill layer (Fig. 10b), due to the low permeability of clay, the clay remains dry in the stress concentration regions, while in the fluid flow regions, the clay is wetted and washed away by the water flow, forming flow channels that are clear to observe. For a gypsum fill layer (Fig. 10c), it can be seen that gypsum is less affected by erosion from the water flow, owing to its good consolidation, and the failure mode of the gypsum band is mainly due to extrusion crushing in the stress concentration regions. This is consistent with the above statement for Fig. 9.

## Discussion

**Figure 11:**
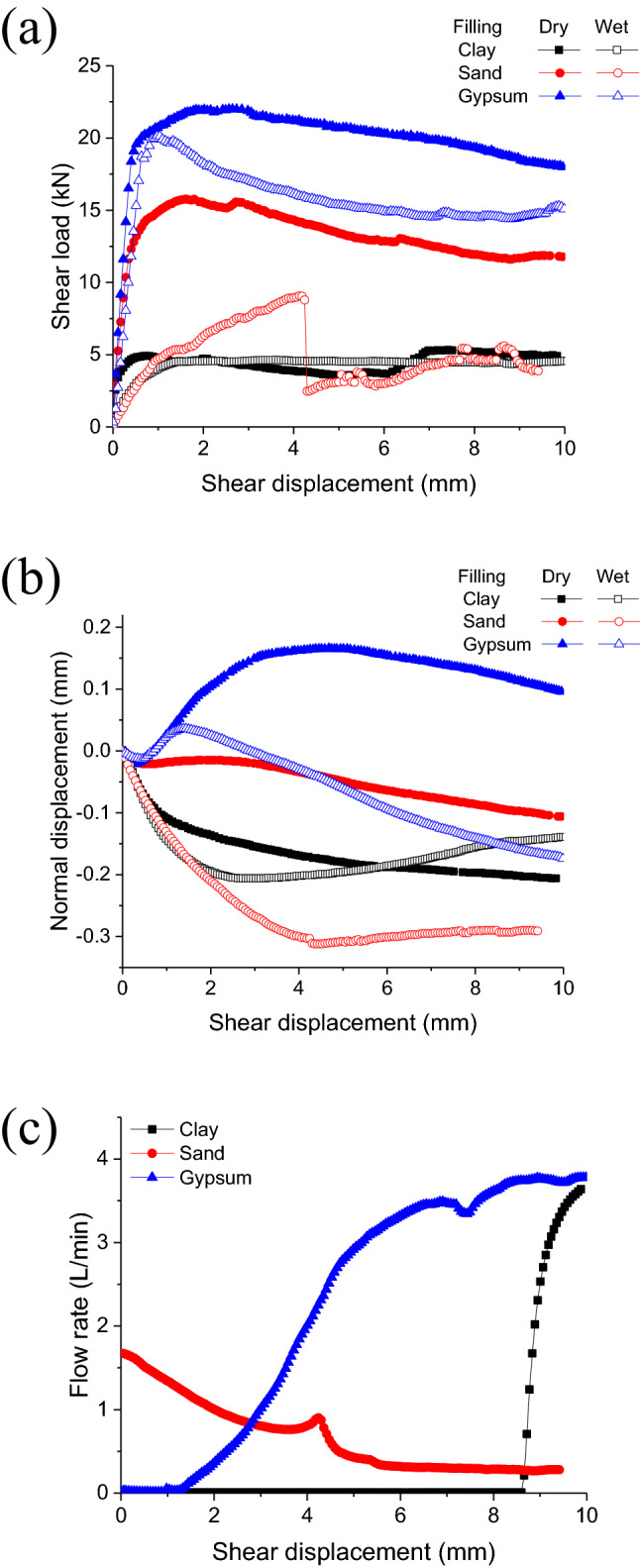
Contrasting curves for different gouge fill materials: (**a**) shear load versus shear displacement, (**b**) normal displacement versus shear displacement, (**c**) flow rate versus shear displacement.

Traditionally, the injected water flow must occur primarily across the gouge, and the effects due to cement-mortar can be neglected in the permeability calculations^46^. Based on the above results, it can be concluded that water injection has a significant weakening effect on the shear strength of fault gouges. However, the weakening mechanism and effect are influenced significantly by gouge material(s). As can be seen from Fig. 11, the weakening effect on shear strength was closely related to the gouge material (Fig. 11a). The gouge permeability determines the range of influence of injected water, which indirectly determines the effective range to which the effective stress is reduced and the gouge material is weakened. On the other hand, the degree of normal deformation during shear is determined by the physical deformation characteristics of the gouge material, which determines the thickness variation of the fill material (Fig. 11b). In the past, Darcy's law was applied to calculate permeability^47^, however, Darcy's law assumes homogeneous flow which, in the present investigation, has been proved to be inaccurate under large displacements (Figs. 10 and 11c). Moreover, it was observed that permeability is closely related to the shear strain under constant load, no matter what the fill material is in the fault gouge. This is also in agreement with Cuss et al.^41^ and Cappa^5^ that the flow paths were localized, and of Faulkner et al.^19^ that permeability is anisotropic during shearing.

**Figure 12:**
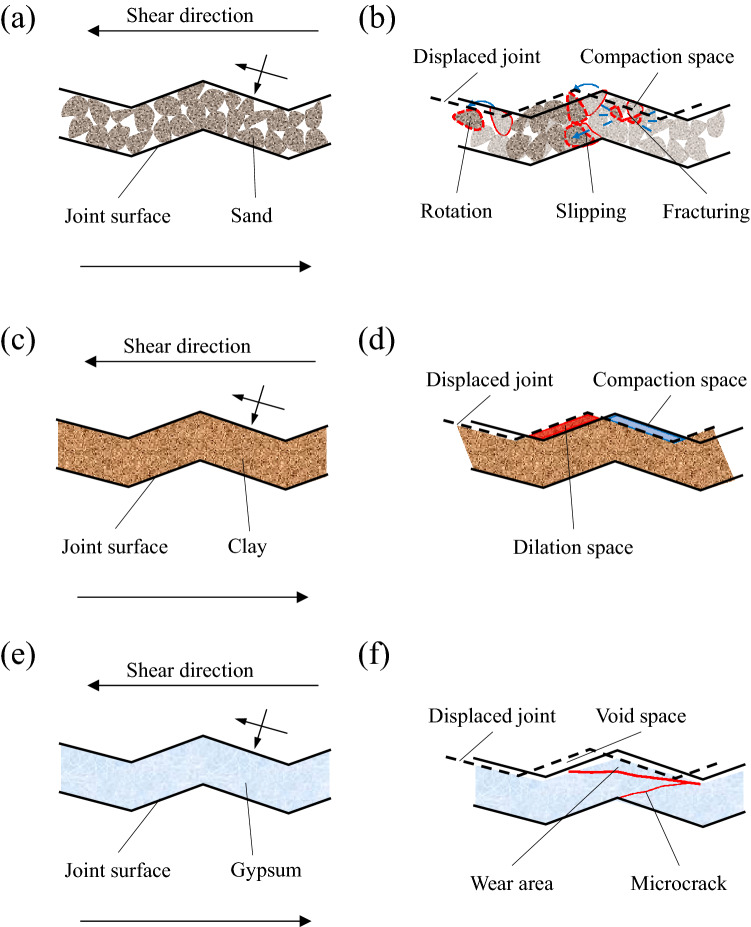
Conceptual scheme of deformation mechanisms of fault gouge, (**a**,**b**) sand, (**c**,**d**) clay, (**e**,**f**) gypsum.

On the basis of previous studies, the conceptual schemes of deformation mechanisms of fault gouges were given for different gouge fill materials. As shown in Fig. 12, the deformation mechanism for different gouge full layers during shearing process is quite different. When the gouge layer is mainly cataclastic rocks (Fig. 12a,b), the cataclastic rocks were re-arranged by rotation and slipping^42^ and may be fractured due the strain and stress localization^48^, caused by shear-enhanced compaction. Porosity and permeability will be decreased and the fluid flow post-shearing may alter the particle size distribution^49^. Clay is soft and is able to adapt to the deformation. It is squeezed from the aperture reduction regions (blue area) to the aperture increasing regions (Fig. 12c,d). When the shear deformation is large, local voids may appear near the water injection hole and the voids tend to be permeated by the water, forming a flow channel^44^. However, owing to the better cementation of gypsum, shear dilatancy will produce a fracture void space and will increase permeability^47^ (Fig. 12e,f). In addition, the gypsum fill layer is damaged at regions of stress concentration, and microcracks will initiate, causing loss of fill continuity, thereby providing a flow path^50^. Thus, not only can deformation evolution influence the hydro-mechanical behavior of faults, but the gouge fill material can be expected to change significantly the fluid transmissivity, flow pattern, and solute transport behavior.

As is illustrated in Fig. 12, grains in the fill band are compacted through rotation and slipping, or fractured to reduce porosity during the shear process. In schematics (12c and 12d) for clay, the clay is squeezed from the aperture reduction regions (blue area) into the aperture increasing regions (red area). When the aperture is large, the clay cannot fill the void, which will lead to water infiltration and the formation of flow channels. In schematics (Fig. 12e,f) for gypsum, the main damage modes were wearing and cracking due to cementation of the gypsum.

## Conclusions

In this study, laboratory tests were conducted to shed light on the physical processes responsible for the weakening of fault gouges resulting from the presence of fluids. It was demonstrated that the strength and deformation of a fault gouge can be weakened by injecting water, despite the presence of different gouge filling materials. Under such conditions, the primary factor that induces instability is the diffusion of pore moisture, which results in a decrease in effective normal stress limit. However, the physical properties of the fault gouge fill material also play an important role in coupling the hydraulic and mechanical aspects, especially under large shear displacements. The weakening effect and the hydraulic characteristics in the process of shear deformation can be entirely different for different types of fault gouge, so the risk of fault instability induced by fluid injection also is different. For fault gouges that contain mainly rough cataclastic particles, which are porous and permeable, injecting water has a significant weakening effect in reducing fault strength, owing to the reduction by pore water pressure of the maximum stress support limit. With low permeability clay-filled gouges, injecting water is mainly associated with weakening of the clay and is focused on the void space regions. In this case, fault stability may not be greatly affected in a short period of time. With regard to the fault gouge with good cementitious properties, in the process of fault slip, water that is injected into fractures or cracks will reduce the effective stress capacity and will decrease the adhesion between fault gouge and the faulted rock, and thereby will reduce the strength of the fault.

## Data Availability

The data of this work will be obtained from the corresponding author (Yixin Liu) if requested.
